# The influence of personal factors, unmet need and service obstacles on the relationship between health service use and outcome after brain injury

**DOI:** 10.1186/s12913-022-07811-y

**Published:** 2022-04-05

**Authors:** David N. Borg, Jennifer Fleming, Joshua J. Bon, Michele M. Foster, Elizabeth Kendall, Timothy Geraghty

**Affiliations:** 1 Griffith University, Menzies Health Institute Queensland, The Hopkins Centre, Brisbane, Australia; 2 Griffith University, School of Allied Health Sciences, Brisbane, Australia; 3 University of Queensland, School of Health and Rehabilitation Sciences, Brisbane, Australia; 4 Australian Research Council Centre of Excellence for Mathematical and Statistical Frontiers, Brisbane, Australia; 5 Queensland University of Technology, School of Mathematical Sciences, Brisbane, Australia; 6 Metro South Health Hospital and Health Service, Division of Rehabilitation, Brisbane, Australia

**Keywords:** Access, Allied health, Rehabilitation

## Abstract

**Background:**

This exploratory study aimed to: (i) examine the relationship between health service use and quality of life, psychological wellbeing, global function and participation after discharge from brain injury inpatient rehabilitation, and (ii) determine the influence of personal factors, unmet need for services and service obstacles on the relationship between service use and these outcomes.

**Methods:**

Using a prospective cohort design, 41 adults with acquired brain injury (median age = 46 years; 71% male; 61% severe traumatic injury) were followed for 6-months after discharge from specialist brain injury inpatient rehabilitation. Service use was continuously recorded and obtained through data linkage methods, focusing on the use of: outpatient medical services, outpatient nursing, outpatient allied health; medical acute services; incidents of re-hospitalization; and transitional rehabilitation service use. Outcome questionnaire measures were completed via telephone, at 6-months after discharge, and included: the EuroQol-5D; Depression Anxiety and Stress Scale, Mayo-Portland Adaptability Inventory and Sydney Psychosocial Reintegration Scale. Data were analyzed in a heterogeneous treatment effects framework, using Bayesian Additive Regression Trees.

**Results:**

There was weak evidence that transitional rehabilitation service use was associated with better psychological wellbeing scores. The posterior probability of lower depression, anxiety and stress scores was .87, .81 and .86, respectively (average treatment effect). There was also weak evidence that re-hospitalization was associated with worse independent living skills scores. The posterior probability of worse scores was .87. However, most re-hospitalizations were due to unavoidable medical complications. We did not find that place of residence at discharge, marital status, unmet need, or service obstacles affected the relationship between service use and the studied outcomes.

**Conclusions:**

This study may highlight the importance of participation in transitional rehabilitation, in the 6-months after discharge from brain injury rehabilitation. Replication in a larger sample size is required to confirm these findings.

**Supplementary Information:**

The online version contains supplementary material available at 10.1186/s12913-022-07811-y.

## Background

Responsive, accessible health systems are critical for outcomes after acquired brain injury (ABI) due to trauma or a neurological event (e.g., stroke) [1–3]. Owing to their protracted recovery involving multiple care transitions and the need for a combination of mainstream and specialist health and rehabilitation services, individuals with moderate-to-severe ABI are likely to be vulnerable to under-performing systems [1, 4]. In Australia, such susceptibility has not been helped by: the historical emphasis on acute and chronic care, rather than rehabilitation; disputes between federal and state governments over funding; and the apparent disregard of rehabilitation services as being an integral part of the healthcare system [5].

After hospital discharge, persons with ABI will ideally continue to access a range of ambulatory services, acute medical services, and other supports. Ambulatory services in brain injury ideally includes specialist rehabilitation and therapeutic services [4, 6]—such as: outpatient day therapy and allied health services, transitional living programs and community rehabilitation services—as well as single discipline, private services [7]. The Australian healthcare system is a complicated arrangement of government and non-government funders, public and private insurance, and a multitude of public and private services. Medicare, the national health insurance system, allows citizens to access public hospitals at no cost, and out-of-hospital medical expenses at no or minimal cost [8]. Those with private health insurance also have access to private hospital and allied health services [8]. This complex arrangement presents a challenge when accessing services, and also when evaluating system performance, particularly when service needs are diverse [9, 10].

Equally challenging is ensuring the optimal mix of services for people with ABI after discharge from hospital. To some extent, the organization of a rehabilitation continuum encourages the standardization, rather than individualization, of treatment and services, in an effort to balance demand with resource limitations [11–13]. However, the response to therapy is not homogenous and increased service use does not necessarily mean better outcome [14–17]. Few studies have directly investigated the relationship between service use and outcome after ABI [15, 17]. These studies suggest there is little-to-no relationship between service use and outcomes of functional independence [15, 17] and cognitive skills [17]. Unfortunately, the relationship between service use and other important outcomes, such as quality of life psychological wellbeing and measures of community integration and participation, has received limited attention.

Adjusting to a new life after hospital discharge can be challenging [18]. Personal factors, such as marital status and place of residence at discharge, can negatively affect outcome, due to their influence on social support and a person’s ability to access services [19, 20]. In addition to place of residence at discharge, the socio-economic disadvantage of a person’s immediate community can influence their ability to access services, with more disadvantaged areas likely to be under-resourced, particularly in terms of private health services, therefore increasing the risk of unmet need [21]. High unmet need for services could also contribute to poorer outcomes during this early transition period [3, 22]. These individuals are also likely to have disproportionate experiences in terms of meeting their needs (i.e., experiences of service obstacles), which could have a detrimental effect on quality of life, psychological wellbeing and integration community [23]. The influence of personal factors, unmet need, and services obstacles on the relationship between health service use and outcome after ABI has not been fully investigated.

This exploratory study aimed to: (i) investigate the relationship between service use and quality of life, psychological wellbeing, global function and participation in the first 6-months after discharge from brain injury inpatient rehabilitation; and (ii) examine the interactive effects of personal factors (i.e., place of residence at discharge, marital status), unmet need, and service obstacles on the relationship between service use and these outcomes. No specific hypotheses were made due to the exploratory nature of the study. However, our expectation was that people discharged to a private residence and those married or in a de facto relationship would have a higher quality of life, lower anxiety and stress, and better global function and participation.

## Methods

### Participants

A convenience sample of 41 adults with ABI were recruited at the time point of discharge from a specialist brain injury inpatient rehabilitation unit, at an Australian tertiary hospital. Participants were recruited between March 2017 and March 2018, as part of a larger project [24]. Forty-nine individuals involved in the larger study did not complete the surveys, and therefore, are not included in the current analysis. There was a lower proportion of people with severe traumatic injuries in the non-responder group (33%) than in the group who completed the surveys (61%; see Supplement 1). Participants were similar on all other sociodemographic, injury and discharge variables (Supplement 1). Participants were included if they met the following criteria: a newly diagnosed ABI, capacity to provide informed consent (or consent by a substitute decision maker on behalf of the participant) and communication skills to complete a telephone survey (or with the assistance from their substitute decision maker). Ethical clearance for the project was obtained from the Princess Alexandra Hospital Human Research Ethics Committee (HREC/16/QPAH/684; SSA/16/QPAH/685) and the Griffith University Human Research Ethics Committee (2016/915). All methods were carried out in accordance with relevant guidelines and regulations. Participants, or their substitute decision makers, provided informed consent.

### Study design

Using a prospective observational cohort design, participants were followed for 6-months, after discharge from specialist inpatient rehabilitation. Health service use was continuously recorded for the 6-month period, with data retrieved via data linkage methods [24]. Telephone surveys were conducted at 6-months, to collect data on a range of outcome measures [24]. All surveys were administered by the same research assistant.

### Outcomes measures

The EuroQol-5D (EQ-5D) was used to capture health-related quality of life [25]. The EQ-5D measures health in five dimensions: mobility, self-care, usual activity, pain/discomfort and anxiety/depression. Participants ascribe a rating to each dimension, using a scale ranging from 1 ‘no’ to 5 ‘extreme problems or unable’. [25] The five responses can be converted to a single utility value, by summing the weights associated with each dimension rating, and then subtracting the summed value from one [26]. For example, dimension ratings of 5–5–5-5-5—the worst ratings on each dimension—correspond to weights of 0.274, 0.203, 0.184, 0.335 and 0.289, respectively [26]. The utility score is calculated as: 1–1.285, giving a utility score of − 0.285. Utility values ranged from − 0.285 ‘worst health’ (i.e., ratings of 5–5–5-5-5) to 1 ‘best health’ (i.e., ratings of 1–1–1-1-1) [26].

Psychological wellbeing was measured using the Depression, Anxiety and Stress Scale short-form (DASS-21) [27, 28]. The DASS-21 contains three, 7-item subscales: depression, anxiety and stress [27, 28]. Participants rate the extent to which each item (statement) applied to them in the past week, using a 4-point scale ranging from 0 ‘never’ to 3 ‘almost always’ [28]. Subscale responses are summed and doubled [27]. The internal consistency of the depression (Cronbach’s alpha [α] = .84), anxiety (α = .76), and stress (α = .89) subscales for our sample was acceptable or greater.

Global function (i.e., functional abilities, psychological adjustment and community reintegration) was evaluated using the Mayo-Portland Adaptability Inventory (MPAI-4) [29]. The 29-item MPAI-4 comprises three subscales: ability, adjustment, and participation—which measure interference with daily or valued activities in key areas [29]. Each item is rated on a 5-point scale, ranging from 0 ‘no problem’ to 4 ‘severe problem’ [29]. Subscale responses were summed, with higher scores indicating greater problems. The internal consistency of the abilities (α = .81), adjustment (α = .82) and participation (α = .86) subscales for our sample was acceptable or greater.

The Sydney Psychosocial Reintegration Scale (SPRS-2) captured the change in participants’ participation after injury, in three domains—occupational activity, interpersonal relationships, and independent living skills [30]. Domains comprise 4-items, with each item rated on a 5-point scale ranging from 4 ‘not at all’ to 0 ‘extreme’. Domain responses were summed, with higher scores indicating less disturbance compared to pre-injury [30]. The internal consistency of occupational activity (α = .80), interpersonal relationships (α = .79), and independent living skills (α = .81) was acceptable or greater.

### Demographic variables and injury characteristics

Participants’ demographic (i.e., age, gender, indigenous status, employment status at the time of injury), injury (i.e., length of stay, traumatic or non-traumatic, severity) and personal context (i.e., marital status, place of residence at discharge) characteristics, and funding support (i.e., whether in receipt of funding through a national injury insurance scheme, or other government funded support) were retrieved from electronic hospital records. The severity of traumatic injuries was classified according standard guidelines [31], as follows: mild traumatic injury (post traumatic amnesia < 24 h; and/or Glasgow Coma Scale 13–15), moderate traumatic injury (post traumatic amnesia > 1 and < 7 days; and/or Glasgow Coma Scale 9–12) or severe traumatic injury (post traumatic amnesia > 7 days; and/or Glasgow Coma Scale 3–8) [31]. When only Glasgow Coma Scale scores were available (*n* = 6) the best score in the first 24 h was used.

Participants completed the Functional Independence Measure at discharge [32]. The 18-item Functional Independence Measure comprises a motor and cognitive subscale. The motor subscale includes 13-items that evaluate functions of self-care, bowel and bladder management and mobility. The cognitive subscale comprises 5-items related to communication and social cognition. Each item is rated on a 7-point scale, ranging from 1 ‘total assistance’ to 7 ‘complete independence’. Items across the two subscales are summed, with higher scores indicating greater independence, and therefore, less physical or cognitive disability [32]. A total score is also calculated. We used the motor subscale in our modelling, as an indicator of a person’s level of physical disability [32]. Cognitive and total scores were used for descriptive purposes only.

The index of relative socio-economic advantage and disadvantage (IRSAD) value of participants’ place of residence at discharge was also included [33]. An area with relatively more disadvantage than advantage could be expected to have poorer healthcare resourcing [21]. IRSAD values were constructed from 2016 Census data, collected by the Australian Bureau of Statistics [33]. An IRSAD value summarizes the relative advantage or disadvantage for a given area (i.e., postcode). Higher values (state deciles) indicate an area with a relatively high incidence of advantage and a relatively low incidence of disadvantage [33].

### Health service use

Participant’s health service use was determined from sources retrieved through the Queensland Health Data Linkage Group. Data sources were: (i) The Queensland Health Non-Admitted Patient Data Collection; (ii) The Queensland Hospital Admitted Patient Data Collection; and (iii) The Emergency Data Collection. The The Queensland Health Non-Admitted Patient Data Collection captured clinic-based appointments at a public hospital or local health network service, for medical specialist, nursing and allied health services [34]. The The Queensland Hospital Admitted Patient Data Collection recorded acute medical service use—i.e., admitted services, including day-admissions—at a hospital service, at public hospitals, licensed private hospitals and private day surgeries [34]. The Emergency Data Collection recorded re-hospitalizations via data from all Queensland Health emergency departments [34]. A re-hospitalization was defined as at least one overnight hospital stay. The type of service used reflects the predominant nature of a service event.

Whether participants accessed a specialist ABI transitional rehabilitation service provided as part of the ABI rehabilitation continuum of care at the study site, after leaving hospital, was also recorded. If attended, transitional rehabilitation involved 10-weeks of intensive rehabilitation in the community, with multi-disciplinary care provided 2–4 times per week. Therapy was delivered on an individual or group basis, and telehealth was available where suitable. Therapies were goal directed, with a focus on return to meaningful activity (e.g., work, study). Family members and/or carers were encouraged to be involved where possible.

### Unmet need for services and service obstacles

Unmet need for services was collected using the statement ‘*Have you needed health care [in the past 6-months] but did not get it’*, to which a yes/no response was recorded [35]. The Service Obstacles Scale captured information about participants’ difficulties in relation to accessing services, knowledge of and availability of resources, and satisfaction with the quality of care [36]. Comprising three subscales: transport (1-item), finances (1-item) and treatment (4-items), Service Obstacles Scale items are rated on a 7-point scale, where responses range from strongly disagree ‘1’ to strongly agree ‘7’ [36]. Responses on the transport and finances subscales were re-categorized to ‘disagree’ 1–3 and ‘agree’ 5–7 for the analysis, due to limited ratings in some categories. The treatment items were summed (range: 4–28), with higher scores indicating less satisfaction with the quality of care received.

### Data analysis

All analyses were performed in R [37]. Missing data were visually inspected (Supplement 2) [38]. Data were assumed missing-at-random, with missing values imputed using predicted mean matching, via the *mice* package [39]. The results were averaged over five imputed datasets.

There were 10 outcome variables: EQ-5D utility scores, and the subscales from the DASS-21 (depression, anxiety and stress), MPAI-4 (ability, adjustment and participation) and SPRS-2 (occupational activity, interpersonal relationships and independent living skills). All models included the predictor variables: outpatient medical service use, outpatient nursing service use, outpatient allied health service use; medical acute service use; re-hospitalization; transitional rehabilitation service use; age; gender; place of residence at discharge; marital status; employment status at the time of injury; length of stay; injury type; comorbidities; funding support; IRSAD value; Functional Independence Measure motor subscale score; unmet need for services; and Service Obstacles Scale transport, finances and treatment scores.

Our primary interest was to investigate the relationship between health service use variables and EQ-5D utility scores, DASS-21 depression, anxiety and stress scores, MPAI-4 ability, adjustment and participation scores, and SPRS-2 occupational activity, interpersonal relationships and independent living skills scores, with a specific focus of the influence of the covariates, including their interactive effects, on the relationship between service use and these outcome variables. To model such a relationship, we used Bayesian Additive Regression Trees (BART), to allow for interactions between service usage variables and the remaining covariates [40]. BART models allow higher-order interactions to be included if substantiated from the data, resulting in a flexible, but parsimonious, regression model [40]. BART models are also useful in this context, as there are many covariates to consider.

To assess the primary relationship between service use and EQ-5D utility scores, and DASS-21, MPAI-4 and SPRS-2 subscale scores, a heterogenous treatment effects approach was taken [41]. Using a heterogenous treatment effects approach, we are able to investigate whether the average response to a particular treatment is mitigated or exacerbated by certain characteristics in subgroups of the subjects, hence the effect is heterogenous across the dataset or population of interest. These ‘treatments’ were represented by the discrete categorization of service use variables, if necessary, at the first quartile. Discrete categories were: outpatient medical (< 2 and ≥ 2 contacts), outpatient nursing (0 and ≥ 1 contact), outpatient allied health (< 2 and ≥ 2 contacts), medical acute (0 and ≥ 1 contact), re-hospitalization (0 and ≥ 1 contact), transitional rehabilitation (0 ‘did not attend’, 1 ‘attended’). Treatment effects are calculated from the model by considering the difference between each participants’ estimated response and their counterfactual response––i.e., their estimated response having received the alternate ‘treatment’. An individuals’ treatment effect is represented by ‘ *τ*_*i*_ ’ in Eq. 1. The values ‘ *y*_*i*_(1) ’ and ‘ *y*_*i*_(0) ’ are the responses to treatment and non-treatment respectively. The response ‘ *y*_*i*_(*z*) ’ will a counterfactual response when ‘ *z* ’ is not the value observed in the data for subject ‘ *i* ’.

Treatment effect equation.

1 $${\tau}_i={y}_i(1)-{y}_i(0)$$

In the Bayesian framework, parameters are treated as random variables with unknown values [42]. The uncertainty of a parameters value is described by the posterior probability distribution which updates the prior distribution with the likelihood once data has been observed (the posterior is proportional to the product of the likelihood and prior distribution). The prior is a statistical distribution that captures the uncertainty in a population parameter before data collection. Using Bayesian modelling methods, we estimated posterior distributions of these treatment effects at the individual level, which can be aggregated to estimate average treatment effects, or aggregated for certain subgroups to estimate conditional average treatment effects. BART estimates the responses ‘ *y*_*i*_(*z*) ’ which depend on many covariates, and the treatment variable. The use of BART models made it is possible to assess whether there was heterogeneity in these effects, as the interactions allow particular subgroups of participants to have varying responses to treatment (i.e., health service use). Where no heterogeneity was observed, effects were averaged over all 41 participants.

In interpreting the results, we focused on marginal effects where the 66% credible interval did not include zero. This standard is one suited to the exploratory nature of the paper, but to emphasise the relatively low threshold, we use the terminology ‘weak evidence’ when referring to the results. Our results are likely to be specific to the population for which data was available but provide direction for future research.

## Results

Participant sociodemographic, injury and discharge characteristics are summarized in Table 1. The median age of participants was 46 years. Participants were generally male (71%), married or in a de facto relationship (46%) and held full- or part-time employment before their injury (68%). These age [43, 44], gender [43, 44] and employment [45] rates are typical of a brain injury population, however, there was a higher proportion of participants who were married or in a de facto relationship than what might be expected [46]. Severe traumatic injuries were most common (61%) and the median length of hospital stay was 47 days—both slightly higher than what might be expected of an Australian sample [46]. As expected, [46] 27% of participants had no comorbidities. Participants were generally discharged to a private residence (85%). Based on a median IRSAD of 6, participants were discharged to locations with slightly greater advantage than about half of the Australian population.

**Table 1:** Participant sociodemographic, injury and discharge characteristics

Variable	*n* = 41
Age (years), median (IQR)	46 (27–59)
Gender	
Male	71%
Female	29%
Marital Status	
Married/de facto	46%
Divorced/separated	17%
Never married	37%
Employment status at the time of injury	
Employed (part- or full-time)	68%
Unemployed	10%
Student	7%
Not in labor force (home duties/child at home)	7%
Retired	7%
Length of hospital stay, median (IQR) days ^a^	47 (30–72)
Injury type	
Non-traumatic	39%
Traumatic—severe ^b^	61%
Comorbidities	
None	27%
Number of comorbidities if present, median (IQR)	2 (1–4)
Funding support ^c^	
No	39%
Yes—National injury insurance funded support	32%
Yes—Other government funded support	22%
Missing	7%
Place of residence at discharge	
Private residence	85%
Interim destination (transitional living unit)	5%
Discharge/transfer to other hospital or rehabilitation facility	10%
SEIFA state IRSAD ^d^	6 (3–9)
Functional Independence Measure at discharge, median (IQR)	
Motor subscale (13–91)	90 (80–91)
Cognitive subscale (5–35)	26 (22–29)
Total (18–126)	114 (106–119)

Percentages may not sum exactly to 100 due to rounding. *IQR* Interquartile range, *IRSAD* Index of relative socio-economic advantage and disadvantage, *SEIFA* Socio-economic indexes for areas

^a^Length of stay calculated from admission to discharge from the rehabilitation unit

^b^All traumatic injuries were classified as severe

^c^Participants entitled to receive health services under a government funded program

^d^Index of relative socio-economic advantage and disadvantage (IRSAD) scores (state decile). Higher scores indicate an area with a relatively high incidence of advantage and a relatively low incidence of disadvantage

Health service use in the immediate 6-months after hospital discharge is shown in Table 2. Table 3 shows unmet need for services, services obstacles and EQ-5D utility, DASS-21 depression, anxiety and stress, MPAI-4 ability, adjustment and participation, and SPRS-2 occupational activity, interpersonal relationships and independent living skills scores at 6-months after hospital discharge.

**Table 2:** Health service use in the immediate 6-months after hospital discharge

Variable	Percentage of participants that accessed services (*n* = 41)	Median (IQR) number of service contacts
Outpatient medical specialist	85%	2 (1–4)
Outpatient nursing	34%	0 (0–1)
Outpatient allied health	90%	3 (0–17)
Medical acute	56%	1 (0–2)
Re-hospitalized ^a^		
Once	22%	–
Twice	5%	–
Transitional rehabilitation	83%	–

*IQR* Interquartile range

^a^Re-hospitalization was considered at least one overnight hospital stay

**Table 3:** Unmet need for services, services obstacles, and health-related quality of life, psychological wellbeing, global function, and participation scores at 6-months after hospital discharge

Variable	*n* = 41
Unmet need for services	10%
Service Obstacles Scale	
Finances ^a^	50%
Transportation ^a^	26%
Treatment (4–28), median (IQR)	12 (8–16)
EuroQol-5D (EQ-5D), median (IQR)	
Utility score (−0.285 to 1)	0.783 (0.697–0.859)
Depression, Anxiety and Stress Scale short-form (DASS-21), median (IQR)	
Depression (0–42)	8 (4–14)
Anxiety (0–42)	4 (2–8)
Stress (0–42)	10 (6–14)
Mayo-Portland Adaptability Inventory (MPAI-4), median (IQR)	
Ability (0–48)	12 (8–17)
Adjustment (0–30)	11 (5–17)
Participation (0–30)	10 (6–14)
Sydney Psychosocial Reintegration Scale (SPRS-2), median (IQR)	
Occupational activity (0–16)	8 (4–11)
Interpersonal relationships (0–16)	12 (9–15)
Independent living skills (0–16)	13 (11–14)

*IQR* Interquartile range

^a^Responses collected on the 1–7 scale and reported as disagree (i.e., 1–3) or agree (i.e., 4–7)

### Health service use and outcome

**Fig. 1:**
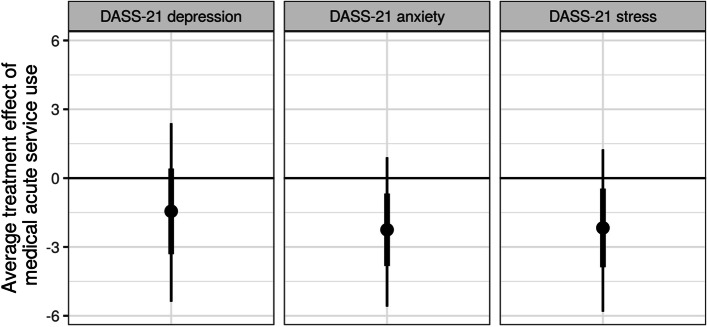
Average treatment effect (averaged across all 41 participants) of one or more medical acute service contacts on DASS-21 depression, anxiety and stress. The posterior probability of lower depression, anxiety and stress scores with one or more medical acute service contacts was .77, .92, and .89, respectively. The posterior mean (circle) is shown with 66% (thick inner line) and 95% (thin outer line) credible intervals

**Fig. 2:**
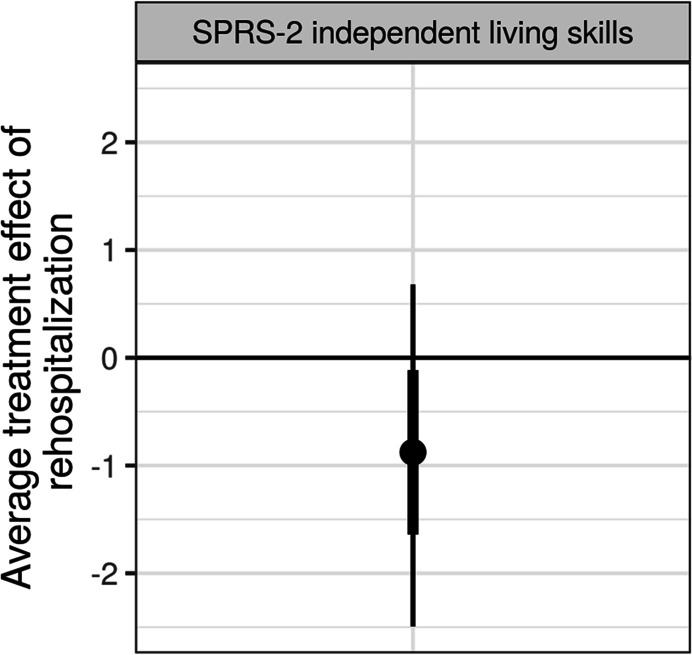
Average treatment effect (averaged across all 41 participants) of re-hospitalization on SPRS-2 independent living skills scores. The posterior probability that re-hospitalization was associated with worse independent living skills scores was .87. The posterior mean (circle) is shown with 66% (thick inner line) and 95% (thin outer line) credible intervals

**Fig. 3:**
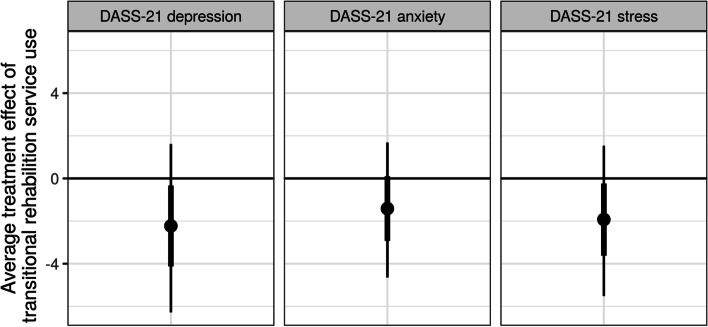
Average treatment effect (averaged across all 41 participants) of transitional rehabilitation service use on DASS-21 depression, anxiety and stress. The posterior probability of lower anxiety, depression and stress scores due to transitional rehabilitation service use was .87, .81, and .86, respectively. The posterior mean (circle) is shown with 66% (thick inner line) and 95% (thin outer line) credible intervals

There was weak evidence that one or more medical acute service contacts was associated with better DASS-21 anxiety and stress scores (Fig. 1). This inverse association also aligned with DASS-21 depression scores, although to a lesser extent (Fig. 1). The posterior probability of lower anxiety, stress and depression scores was .92, .89, and .77, respectively (i.e., average treatment effect). There was weak evidence that re-hospitalization was associated with worse (lower) SPRS-2 independent living skills scores (Fig. 2). The posterior probability of lower living skills scores if re-hospitalized was .87. There was weak evidence that transitional rehabilitation service use was associated with better DASS-21 depression, anxiety and stress scores (Fig. 3). The posterior probability of lower depression, anxiety and stress scores was .87, .81 and .86, respectively.

### Interactive effects on the relationship between health service use and outcome

There was no evidence of heterogenous treatment effects for any health service use variable; and therefore, no evidence that place of residence at discharge, marital status, unmet need, or service obstacles affected the relationship between health service use and quality of life, psychological wellbeing, global function or participation. Therefore, we did not find any indication that the average response to a particular service use was either mitigated or exacerbated by certain characteristics in subgroups of the participants. The BART models also did not indicate that age, gender, employment status, length of stay, injury type, comorbidities, funding support, IRSAD value, Functional Independence Measure motor subscale score, unmet need, or transport, finances and treatment obstacles had an effect on the studied outcomes.

## Discussion

There was weak evidence that transitional rehabilitation service use was associated with better psychological wellbeing, and re-hospitalization with poorer independent living skills. There was also weak evidence that one or more medical acute service contacts (i.e. admitted services, including day-admissions) was associated with higher levels of psychological wellbeing. We did not find evidence that personal factors, unmet need or service obstacles affected the relationship between health service use and the outcomes of quality of life, psychological wellbeing, global function, and participation. It is possible that there are subgroups in the studied population that are susceptible to poorer scores on these outcome measures after discharge from inpatient brain injury rehabilitation, but a larger sample size would be required to identify individuals in these subgroups. Our findings may highlight the importance of transitional rehabilitation and other similar community rehabilitation services, particularly those that target psychological wellbeing, in the 6-months after discharge from brain injury rehabilitation.

In agreement with previous work [15] there was only weak evidence that health service use was related to outcome after brain injury. Stronger evidence is required to validate our findings. A control group where services are required but cannot be accessed would shed further light on the findings. While unfortunate, COVID-19 related service impacts—for example, outpatient service closures to accommodate additional acute hospital space—could make it possible to study the effects of being unable to access required services on outcomes, such as quality of life, psychological wellbeing and global function. Surprisingly, only four participants in the study reported unmet need for services. It is possible that unmet need in the study sample was low because most participants (83%) attended a transitional rehabilitation service. However, the four individuals who reported unmet need also attended transitional rehabilitation. Based on the available data it is not possible to identify any pattern related to the reporting of unmet need (Supplement 3).

Engagement with a specialist post-acute rehabilitation service after brain injury has been shown to reduce disability [47], and improve participation [47, 48] and psychological wellbeing [48, 49]. Our findings may provide further evidence that specialist transitional rehabilitation service use positively affects psychological wellbeing. Given the role that psychological status has on function after brain injury, this may have important implications for the approach to treatment, depending on the level of symptoms [50]. Psychological resilience has been found to be uniquely important to participation in a range of activities for many years following brain injury [51]. More importantly, constructs such as psychological flexibility have been found to impact the ability to benefit from treatment [52]. If treatment, such as transitional rehabilitation, can positively influence psychological wellbeing, perhaps by supporting psychological flexibility, then subsequent treatments may be even more effective [48]. Further, there may be a reduction in long-term mental ill-health associated with ABI. Thus, the ability to influence psychological wellbeing may have multiple beneficial impacts that extend beyond outcomes of psychological wellbeing.

We found weak evidence that re-hospitalization was associated with poorer independent living skills. This finding could raise the importance of early access to health services that target independent living skills [53]. Re-hospitalisations come at a significant cost to the healthcare system [54]. Between 2012 and 2015, the cost of an acute admitted patient in Queensland hospitals was estimated to range from AUD $4700 to $5600 [55]. However, of the 13 incidence of re-hospitalisation, 85% (11/13) were due to unavoidable medical complications (i.e., headaches or seizures), rather than due to causes directly affected by independent living skills. As such, we are unable to explain the association, and this finding should be interpreted with caution. The BART model did not identify injury severity, physical disability (i.e., Functional Independence Measure motor scores), or any other factor included in the model that might be associated with rehospitalization and independent living—including the interaction with re-hospitalization—as being important/associated with independent living skills.

### Study limitations

The primary limitation of this study was the sample size. We did not find any indication that age, gender, employment status, length of stay, injury type, comorbidities, funding support, IRSAD value, Functional Independence Measure motor scores, unmet need, or any service obstacle were important predictors of quality of life, psychological wellbeing, global function or participation. However, this could be due to the small sample size. Participants in the study were recruited from a single hospital, and where relevant, accessed the same transitional rehabilitation service. Therefore, our results are by no means generalizable. The absence of outcome measures at discharge to serve as a baseline may be considered a limitation. However, this would only be of relevance for the EQ-5D and DASS-21, as capturing the MPAI-4 and SPRS-2 and at discharge does not conceptually make sense, as most questions assume the person is discharged and in the community. Recruitment for this study coincided with the introduction of new government funded, disability-related insurance scheme. It is possible that the benefits of these systemic changes designed to facilitate better access and outcomes were not yet being fully realized.

## Conclusion

Findings from our study may highlight the importance of transitional rehabilitation services, after discharge from specialist inpatient brain injury rehabilitation. We did not find any evidence that personal factors, unmet need, or service obstacles, affected the relationship between health service use and quality of life, psychological wellbeing, global function, and participation. Identifying the factors that regulate the relationship between service use and outcomes (e.g., psychological wellbeing, global function) after brain injury, particularly during the transition from hospital-to-home, would provide important information to policy makers and should be a future research priority.

## Supplementary Information

**Additional file 1.**

**Additional file 2.**

**Additional file 3.**

## Data Availability

The R code used to generate the analyses in the manuscript are available upon request from the corresponding author. A limited, non-identifiable dataset will also be made available upon request.

## Abbreviations

ABI: Acquired brain injury
BART: Bayesian Additive Regression Trees
DASS-21: Depression, Anxiety and Stress Scale short-form
EQ-5D: EuroQol-5D
IRSAD: Index of relative socio-economic advantage and disadvantage
MPAI-4: Mayo-Portland Adaptability Inventory
SPRS-2: Sydney Psychosocial Reintegration Scale
