# Supplementary material for: The influence of personal factors, unmet need and service obstacles on the relationship between health service use and outcome after brain injury
Source: BMC Health Serv Res. 2022 Apr 5;22:445. doi: 10.1186/s12913-022-07811-y (PMC8980503; doi:10.1186/s12913-022-07811-y)
Supplement: Supplementary file 1 — Additional file 1. [file 12913_2022_7811_MOESM1_ESM.docx]

**Supplementary 1.** Patient sociodemographic, injury and discharge characteristics of participants who did and did not complete surveys.

| Variable | Survey (n=41) | Non-survey (n=49) | *p*-value |
| --- | --- | --- | --- |
| Age (years), median (IQR) | 46 (27–59) | 42 (24–58) | .42 |
| Gender |  |  |  |
| Male | 71% | 71% | 1 |
| Female | 29% | 29% |  |
| Marital Status |  |  | .50 |
| Married/defacto | 46% | 39% |  |
| Divorced/separated | 17% | 10% |  |
| Never married | 37% | 45% |  |
| Missing | 0% | 6% |  |
| Indigenous status |  |  | .13 |
| Aboriginal, but not Torres Strait Islander | 0% | 8% |  |
| Neither | 100% | 92% |  |
| Employment status at the time of injury |  |  | .79 |
| Employed (part- or full-time) | 68% | 69% |  |
| Unemployed | 10% | 14% |  |
| Student | 7% | 8% |  |
| Not in labor force (home duties/child at home) | 7% | 2% |  |
| Retired | 7% | 6% |  |
| Length of hospital stay, median (IQR) days † | 47 (30–72) | 43 (28–66) | .47 |
| Injury type |  |  | **.015 *** |
| Non-traumatic | 39% | 59% |  |
| Traumatic—mild | 0% | 2% |  |
| Traumatic—moderate | 0% | 4% |  |
| Traumatic—severe | 61% | 33% |  |
| Missing | 0% | 2% |  |
| Comorbidities, median (IQR) | 2 (0–3) | 2 (1–3) | .55 |
| Funding support ‡ |  |  | .60 |
| No | 39% | 39% |  |
| Yes—National injury insurance funded support | 32% | 18% |  |
| Yes—Other government funded support | 22% | 20% |  |
| Missing | 7% | 22% |  |
| Place of residence at discharge |  |  | 1 |
| Private residence | 85% | 84% |  |
| Interim destination (transitional living unit) | 5% | 6% |  |
| Discharge/transfer to other hospital/rehab. facility | 10% | 10% |  |
| SEIFA state IRSAD § | 6 (3–8) | 7 (4–9) | .56 |
| FIM motor discharge score (13–91), median (IQR) | 90 (80–91) | 89 (80–91) | .36 |

Note. Percentages may not sum exactly to 100 due to rounding. Continuous or count variables were analyzed using a Mann-Whitney-Wilcoxon test, and categorical variables using Pearson’s chi-square test. A Monte Carlo simulated *p*-value (2,000 replicates) was used for Pearson’s chi-square tests when cell counts were less than five. FIM = Functional independence measure; IQR = Interquartile range; IRSAD = Index of relative socio-economic advantage and disadvantage; SEIFA = Socio-economic indexes for areas

* indicates a statistical difference between groups.

† Length of stay calculated from admission to discharge from the rehabilitation unit.

‡ Participants entitled to receive health services under a government funded program.

§ A higher IRSAD value indicates an area with a relatively high incidence of advantage and a relatively low incidence of disadvantage.
