# Supplementary material for: The influence of personal factors, unmet need and service obstacles on the relationship between health service use and outcome after brain injury
Source: BMC Health Serv Res. 2022 Apr 5;22:445. doi: 10.1186/s12913-022-07811-y (PMC8980503; doi:10.1186/s12913-022-07811-y)

**Supplementary 2.** Graphical summary of missing data (A) and number of missing values in each variable (B). Data were assumed missing at random.


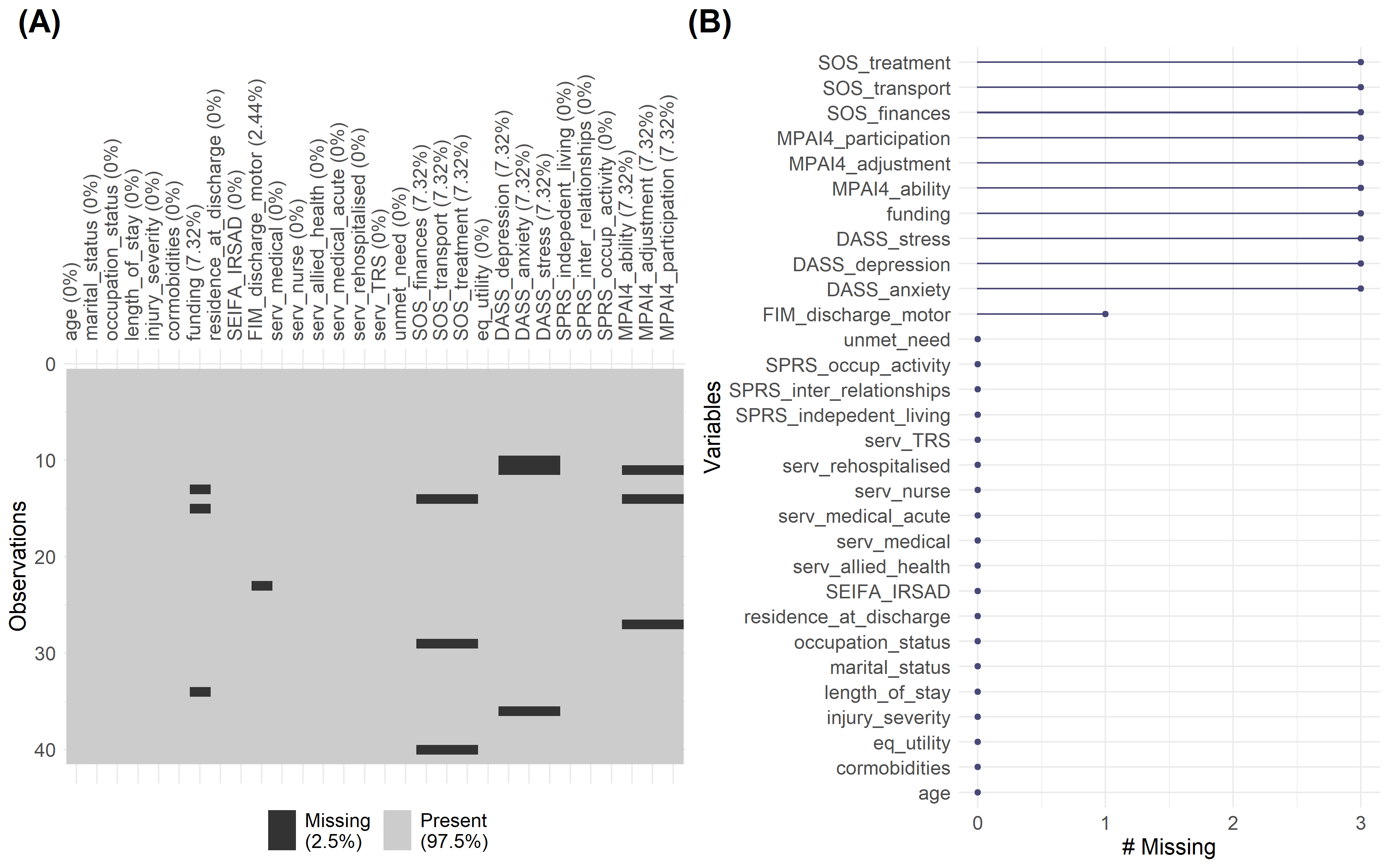

Supplement: Supplementary file 2 — Additional file 2. [file 12913_2022_7811_MOESM2_ESM.docx]
