# Supplementary material for: The influence of personal factors, unmet need and service obstacles on the relationship between health service use and outcome after brain injury
Source: BMC Health Serv Res. 2022 Apr 5;22:445. doi: 10.1186/s12913-022-07811-y (PMC8980503; doi:10.1186/s12913-022-07811-y)
Supplement: Supplementary file 3 — Additional file 3. [file 12913_2022_7811_MOESM3_ESM.docx]

**Supplementary 3.** Participants who reported unmet need for services.

| Variable | Participant A | Participant B | Participant C | Participant D |
| --- | --- | --- | --- | --- |
| Gender | Female | Male | Male | Male |
| Injury type | Non-traumatic | Traumatic (severe) | Traumatic (severe) | Traumatic (severe) |
| Funding support | None | Other | NIIFS | Missing |
| Health service use |  |  |  |  |
| Outpatient medical specialist | 4 | 1 | 3 | 3 |
| Outpatient nursing | 2 | 0 | 0 | 0 |
| Outpatient allied health | 17 | 30 | 1 | 1 |
| Medical acute | 2 | 2 | 0 | 2 |
| Re-hospitalized | No | Yes | No | No |
| Transitional rehabilitation | Yes | Yes | Yes | Yes |
| Service Obstacles Scale |  |  |  |  |
| Finances | No | Yes | No | Yes |
| Transportation | Yes | Yes | Yes | Yes |
| Treatment (4–28) * | 14 | 26 | 16 | 18 |
| Quality of life (EuroQol-5D-5L) † |  |  |  |  |
| Utility score | 0.783 | 0.142 | 0.922 | 0.846 |
| Psychological wellbeing (DASS-21) ‡ |  |  |  |  |
| Depression | 18 \|\| | 20 \|\| | 2 | 8 |
| Anxiety | 2 | 10 \|\| | 8 § | 0 |
| Stress | 14 | 16 § | 16 § | 16 § |
| Global function (MPAI-4) ‡ |  |  |  |  |
| Ability | 15 | 18 | 8 | 26 |
| Adjustment | 18 | 21 | 2 | 18 |
| Participation | 14 | 19 | 0 | 12 |
| Participation (SPRS-2) † |  |  |  |  |
| Independent living skills | 13 | 11 | 14 | 14 |
| Interpersonal relationships | 7 | 4 | 13 | 9 |
| Occupational activities | 6 | 1 | 12 | 5 |

Note. Participant D missing funding support data. DASS-21 = Depression, Anxiety and Stress Scale short-form; MPAI-4 = Mayo-Portland Adaptability Inventory; NIIFS = National injury insurance funded support; SPRS-2 = The 5-point Sydney Psychosocial Reintegration Scale

* Higher scores indicate less satisfaction with the quality of care received

† Higher scores/values indicate better function

‡ Lower scores indicate better function

§ Mild symptoms

|| Moderate symptoms
